# Potential Predictors of Long COVID in Italian Children: A Cross-Sectional Survey

**DOI:** 10.3390/children11020221

**Published:** 2024-02-09

**Authors:** Marco Schiavo, Paola Di Filippo, Annamaria Porreca, Giovanni Prezioso, Greta Orlandi, Nadia Rossi, Francesco Chiarelli, Marina Attanasi

**Affiliations:** 1 Pediatric Allergy and Pulmonology Unit, Department of Pediatrics, University of Chieti-Pescara, 66100 Chieti, Italy; marco.schiavo@studenti.unich.it (M.S.); difilippopaola@libero.it (P.D.F.); gprezsioso@hotmail.it (G.P.); gretaandrea.orlandi@studenti.unich.it (G.O.); nadiarosit@yahoo.it (N.R.); chiarelli@unich.it (F.C.); 2 Department of Economic Studies, University of Chieti, 66100 Chieti, Italy; annamaria.porreca@unich.it

**Keywords:** long COVID syndrome, risk factors, comorbidities

## Abstract

Background: Identifying predictive factors of long COVID syndrome (LCS) is essential to preventing and managing this condition. We investigated the prevalence, symptoms, and risk factors of LCS in a cohort of Italian children and adolescents. Methods: We carried out a cross-sectional survey on demographic characteristics and clinical data related to COVID-19 phase and LCS in a cohort of children and adolescents, sending a questionnaire by using the PEDIATOTEM platform. Results: The prevalence of LCS was 25% (99/396). The most frequent symptoms of LCS included nasal congestion, diarrhea, headache, and fatigue. We found no association between demographic data (gender, age, and ethnicity) and LCS. Additionally, we showed that patients with concurrent allergic rhinitis, atopic dermatitis, respiratory disease, gastrointestinal disease, and rheumatologic disease had a higher risk of LCS than patients without those comorbidities. Patients experiencing fatigue, muscle, and abdominal pain in COVID-19 showed a higher risk of LCS than patients complaining of other symptoms. We found no association between vaccination and LCS. Conclusions: Specific comorbidities or symptoms during acute illness were identified as being risk factors for LCS. Understanding which are the risk factors for LCS could yield a clearer picture of its pathogenesis.

## 1. Introduction

There is growing recognition that the Coronavirus Disease 2019 (COVID-19) pandemic resulted in long-term symptoms in a significant proportion of the population. Long COVID syndrome (LCS) is a condition characterized by the persistence of signs and symptoms after recovery from an acute Severe Acute Respiratory Syndrome Coronavirus 2 (SARS-CoV-2) infection [1]. To date, the estimated prevalence of LCS is up to 42% of infected patients in general [2] and 25.2% of children and adolescents [3]. In December 2020, the United Kingdom National Institute for Health and Care Excellence defined LCS as signs and symptoms that continue or develop after acute COVID-19: this definition includes both ongoing symptomatic COVID-19 (from 4 to 12 weeks) and post-COVID-19 syndrome (12 weeks or more) [4].

More recently, the World Health Organization defined LCS in children and adolescents according to the Delphi-consensus process as the presence of symptoms lasting more than 12 weeks from acute infection [5].

The Italian National Institute of Health defined LCS as the persistence of symptoms 4 weeks after acute COVID-19 [6]. Despite a lower acute infection severity in children than in adults, the COVID-19 pandemic had a significant negative impact on children’s psychosocial health and relational and social well-being [7]. The most common symptoms of LCS in children were somatic, but the clinical presentation and timing of exacerbation and resolution were heterogeneous [7].

LCS diagnosis has become increasingly difficult as it is characterized by several symptoms, ranging from malaise, fatigue, and muscular fatigue to autonomic dysfunction [8]. Therefore, the most frequent symptoms of LCS mimic chronic fatigue syndrome/myalgic encephalomyelitis and dysautonomia, implying a decrease in daily function and quality of life [9].

Initially, it was unclear whether these symptoms were related to SARS-CoV-2 infection or due to the consequences of the pandemic (increased stress, social isolation, restrictions, uncertainty, and lockdown) [10].

Successively, a nationwide Danish cross-sectional study comparing 10.997 children with confirmed acute COVID-19 and 33.016 matched controls found that children with a SARS-CoV-2 infection had higher odds of LCS [11], suggesting that LCS could be specifically related to the previous SARS-CoV-2 infection. In a large meta-analysis, Lopez-Leon et al. [3] confirmed that the most frequent manifestations of LCS in children and adolescents were neuropsychiatric: mood symptoms (sadness, tension, anger, anxiety, and depression), fatigue, and sleep disorders. Moreover, a strong association between sleep disturbance and anxiety and stress was already demonstrated [12]. Other frequent symptoms were headache (7.8% of subjects), respiratory symptoms (7.6%), nasal congestion (7.5%), orthostatic intolerance (6.9%), cognition symptoms (6.3%), loss of appetite (6.1%), and exercise intolerance (5.7%) [3].

Recently, the risk factors associated with the development of LCS were investigated. Studies on adults identified female sex, severity of initial respiratory disease or overall acute COVID-19 infection, and length of hospitalization for COVID-19 infection as risk factors for CSF [9]. A patient’s diet, alcohol use, smoking behaviors, amount of exercise prior to acute COVID-19 infection, and prior sleep patterns may also contribute to the development of LCS [13]. However, little is known about the risk factors for LCS in children and adolescents. 

In a retrospective study including 237 children and adolescents with a mean age of 6.5 years and a previous COVID-19 infection, older age and obesity were risk factors for LCS, developed in 17% of the sample. The authors also observed that respiratory symptoms occurred early in LCS, while neurocognitive symptoms became more common the longer the symptoms persisted [14].

The underlying pathophysiological mechanism of LCS is still unclear. It was hypothesized that deconditioning due to inactivity, particularly in case of severe disease, could be the cause of LCS. However, vaccination status and other social factors of health (such as socioeconomic status, access to healthcare, and transportation, etc.) may also play roles [9].

Regarding vaccination status for COVID-19 at the time of initial COVID-19 infection, a recent survey on 62,000 participants by the Center for Disease Control and Prevention found a higher number of self-reported LCS cases in states with the lowest vaccination rates (Wyoming, Mississippi, Louisiana, and Idaho) compared to states with the highest vaccination rates (Rhode Island, Maine, Vermont, and Massachusetts (16.1–21% vs. 6.7–12.8%) [15].

COVID-19 vaccination decreases the risk of severe illness, but its impact on the prevalence of LCS is still unclear. A systematic review found that COVID-19 vaccination was associated with a lower risk of LCS [16]. However, studies were mostly conducted in the short-term period after vaccination and further studies are needed to clarify the long-term role of the vaccine in the development of LCS. Understanding the relationship between LCS and vaccination status is essential to aid with ongoing education and recommendations.

Although LCS has been widely investigated in adults, little is known about its clinical course and associated predictive factors in children and adolescents. The identification of predictors of LCS in children is crucial for managing the healthcare plans of patients and preventing this condition. According to the European Respiratory Society, the risk factors for LCS are female sex, age, and the number of symptoms experienced at infection onset instead of the acute illness severity [3,17,18,19]. However, further detailed larger studies are needed to better understand which risk factors might affect LCS in children. Hence, the study aim was to investigate the symptoms of acute COVID-19 and LCS and to identify predictive factors of LCS in a cohort of Italian children and adolescents.

## 2. Materials and Methods

We carried out a cross-sectional survey in the late phase of the pandemic. The data were collected in Abruzzo, a region in central Italy, from September 2022 to January 2023. Patients > 18 years old or with severe neurocognitive disability were excluded, since this would have not allowed for a proper assessment of the signs and symptoms included in the survey. Caregivers were interviewed about their child’s health using a questionnaire developed by the “International Severe Acute Respiratory and emerging Infection Consortium” (ISARIC) for COVID-19 follow-up in children working groups, informed by a wide range of global stakeholders with expertise in infectious diseases, critical care, pediatrics, epidemiology, allergy-immunology, respiratory medicine, psychiatry, psychology, and methodology and patient representatives. In particular, after obtaining approval by email, we received, in February 2022, the Pediatric Follow-up form (https://isaric.org/research/covid-19-clinical-research-resources/paediatric-follow-up/, accessed on 6 February 2022), ISARIC Global Pediatric Follow-up Case Control Survey, version 1.4 translated into Italian.

The ISARIC Global Pediatric Follow-up Case Control Survey is a survey that aims to investigate the prevalence and risk factors of post-COVID-19 conditions in children [20]. Osmanov et al. [18] carried out a prospective cohort study including children (≤18 years old) admitted to hospital with confirmed COVID-19 and used the ISARIC Global Follow-up Protocol. The authors found that a quarter of children experienced persistent symptoms months after hospitalization with acute COVID-19 infection, with almost 1 in 10 experiencing multisystem involvement. An older age and allergic diseases were associated with a higher risk of persistent symptoms at follow-up. Similarly, Funk et al. [21] identified factors associated with reporting persistent, new, or recurring health problems in their cohort study of 1884 SARS-CoV-2–positive children with 90-day follow-up. The survey evaluates physical and psychosocial health and well-being, and impact on daily functioning, behavior, relationships, and daily living [22]. It describes data on demographics, pre-existing comorbidities, acute severity, information on the acute phase of the disease (symptoms, comorbidities, and clinical outcomes), and severity (hospital admission, pediatric intensive care, and oxygenation). Additionally, it collects data on re-hospitalizations, parental perception of changes in their child’s emotional and behavioral status, including reasons for observed changes (direct or indirect impact of COVID-19 or both), persisting symptoms at follow-up assessment, and overall health condition compared to prior to the index case of SARS-CoV-2 diagnosis, and mortality [22]. The survey also included the EuroQol EQ-5D-Y-3L test [22] as a version for younger respondents, which we decided to distribute later by contacting the families via a phone call. However, the findings on the EuroQol session will be shown in a subsequent study. Therefore, we first used a modified version of ISARIC without the EuroQol session. 

We defined healthy children or adolescents without previous COVID-19 diagnosis as “controls”. In this study, we aimed to investigate the potential predictors of LCS among Italian children or adolescents who lived in the Abruzzo region. Additionally, a description of how the experience of Italian children or adolescents after COVID-19 could impact their health status is described in our ongoing study. We recruited primary care pediatricians (PCPs) who worked in Abruzzo by using a specific platform, PEDIATOTEM (PEDIATOTEM (PCPs), Azienda LVIIIER srl; https://www.pediatotem.it/PediaTotem_sito/index.html, accessed on 3 September 2022). PEDIATOTEM is an application which was designed to improve the quality of communication between pediatricians and their patients. This app offers a messaging system that allows patients to send photos and share communications with their doctor, saving them time and money. He PCPs received a notification that included a cover letter about the aim and methods of the survey, and how to join the study. The PCPs’ recruitment lasted at least 3 weeks. Non-respondent PCPs were contacted by PEDIATOTEM notification with one reminder after 1 week from the first invitation to increase participation. After obtaining the PCPs’ consent to join the study, the PCPs provided the ISARIC questionnaire to the children’ caregivers (aged 6–18 years) by using the PEDIATOTEM platform. We considered that each parent/caregiver/guardian for both their own and children’s participation indirectly provided us with consent as soon as they decided to fill out the survey. The survey was preceded by the cover letter where the study procedures were explained. The study was conducted in accordance with the Declaration of Helsinki and approved by the Regional Ethics Committee of Abruzzo (C.Et.R.A.).

### Statistical Analysis

The data were analyzed using descriptive statistics appropriate for the nature of the variables. Continuous variables were presented with the number of observations, first quartile (Q1), second quartile (median), and third quartile (Q3). Categorical data were presented as absolute frequencies (*n*) and percentages (%). Pearson’s Chi-squared test (for cell frequency *n* ≥ 5) and Fisher’s exact test (for cell frequency *n* < 5) were used to investigate the associations between categorical variables. For continuous variables, differences between groups were assessed using the Mann U Whitney test. The normality assumption was assessed using the Shapiro–Wilk test. The simple logistic regression model was used to investigate the associations of the demographic data, comorbidities, and COVID-19 symptoms (as exposures) with LCS (as outcome). All the association measures were expressed as odds ratios (OR) and 95% confidence intervals (CI). All *p*-values were two-tailed, and a *p*-value of ≤0.05 indicated a statistically significant association.

All statistical analyses were performed using the R statistical environment (version 4.3 R Foundation for Statistical Computing, Vienna, Austria).

## 3. Results

Ten pediatricians joined the study. The survey questionnaire was sent to 4.823 caregivers and their children. The response rate of the survey was 12.4%, as 600 participants responded to the questionnaire.

**Figure 1:**
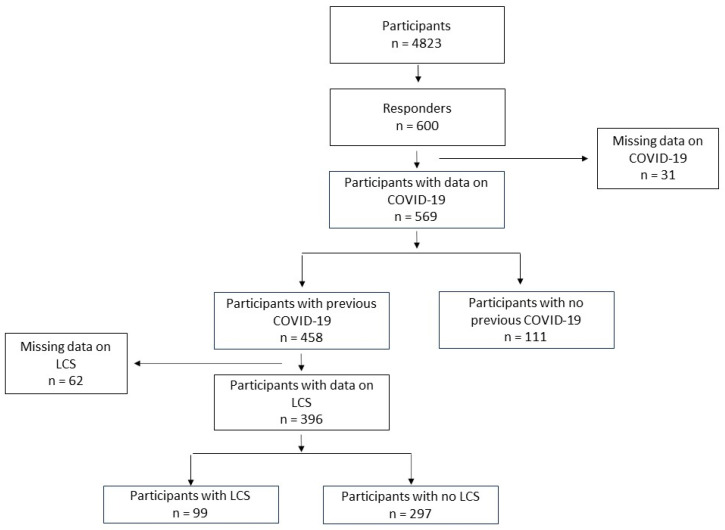
Flow-chart of the study. LCS: Long COVID syndrome; COVID-19: COronaVIrus Disease of 2019.

The flow-chart of the study is shown in Figure 1.

Finally, 569 participants completed the survey questionnaire: 458 patients with a previous COVID-19 diagnosis (cases) and 111 controls. The general characteristics of the participants with or without previous COVID-19 (controls) are shown in Table S1 (Supplementary Material). Two hundred and ninety-seven completely recovered and complained of no symptoms after COVID-19 infection. Contrarily, 99/396 cases (25.0%) reported Long COVID symptoms: 31 patients (31.3%) with symptoms lasting 4–12 weeks and 68 patients (68.7%) with symptoms lasting more than 12 weeks. 

### 3.1. General Characteristics of the Study Population

The Long COVID group consisted of 51 females (51.5%). The mean age of the patients with LCS was 9 (2.7) years. Importantly, 15 patients (15.2%) were adolescents (>12 years of age). In total, 71 patients (91%) were Caucasian and 7 (9%) belonged to other ethnic groups. Nasal congestion and diarrhea (34.3%) were equally the most frequently reported symptoms of LCS, followed by headache (23.2%), fatigue (21.2%), loss of appetite (13.1%), insomnia (12.1%), abdominal pain (10.1%), cough (8.1%), vision problems (7.1%), and confusion with impaired concentration (5.0%). During COVID-19, those patients showed more frequently fever (58.6%), headache (49.5%), rhinorrhea (38.4%), sore throat (38.4%), cough (33.3%), muscle pain (32.3%), and fatigue (29.3%). Vomiting, abdominal pain, loss of taste, diarrhea, confusion, chest pain, and brain fog were less frequently reported. Additionally, allergic rhinitis (25.3%), atopic dermatitis (13.1%), gastrointestinal problems (vomiting, diarrhea, and abdominal pain) (10.1%), food allergies (9.1%), respiratory diseases (5.0%), rheumatologic diseases (3.0%), and anxiety (3.0%) were more frequently mentioned as comorbidities. 

Most of the children of our sample were vaccinated (61.9%) with two doses.

### 3.2. Associations of Demographic Data, Comorbidities, and COVID-19 Symptoms with LCS

We found no association between demographic data and LCS: female (OR (CI 95%) 1.03 (0.65, 1.62)), adolescents (1.43 (0.72, 2.74)), and Caucasian ethnicity (1.46 (0.64, 3.82)). It was noteworthy that we found significant associations of the presence of allergic/respiratory, rheumatologic, and gastrointestinal comorbidities with LCS. In particular, patients with concurrent allergic rhinitis, atopic dermatitis, and food allergies showed a higher risk of LCS than patients without those comorbidities (5.50 (2.83, 10.90); 4.29 (1.81, 10.50)); (4.09 (1.46, 12.00), respectively). Similarly, patients with concurrent respiratory, gastrointestinal, and rheumatologic diseases showed a higher risk of LCS than children without those comorbidities (7.45 (1.51, 58.7)); (7.97 (2.55, 30.6); (21.51 (1.10, 420.12), respectively) (Table 1).

Regarding symptoms during the acute illness, we observed that children with fatigue, muscle, and abdominal pain showed a higher risk of LCS (2.73 (1.57, 4.74)); (2.01 (1.20, 3.34)); (3.14 (1.03, 9.59), respectively) (Table 2). Lastly, we also observed that vaccine administration was not associated with LCS (1.49 (0.89, 2.53)) (Table 1).

## 4. Discussion

Our study identified the prevalence, clinical characteristics, and predictive factors of LCS in a group of 396 Italian children and adolescents. We found that participants with the concurrent presence of allergy, respiratory, gastrointestinal, and rheumatologic diseases showed a higher risk of developing LCS than participants without those comorbidities. In addition, we observed that females, adolescents, and Caucasians had a higher risk of developing LCS than males, children, and other ethnicities, although this was not statistically significant.

Overall, 25.0% of our study population developed LCS. In the literature, the prevalence of LCS in adults was higher than in children. In a recent retrospective longitudinal study, including 436 Italian adult inpatients and outpatients previously diagnosed with COVID-19, the authors found LCS in 71.8% of patients [23]. Similarly, in a cohort of 1655 adults, LCS developed in 76% of subjects 6 months after hospitalization for COVID-19 [24]. A recent meta-analysis with 1,680,003 non-hospitalized and hospitalized COVID-19 patients across the world showed a global prevalence of LCS of 42%, although the patients were mostly adults [2].

In children, a lower prevalence of LCS was observed than in adults. The prevalence of LCS in our study was consistent with a meta-analysis including 80,071 children and adolescents, with a prevalence of 25.2% [3]. Similarly, another meta-analysis including 12,424 children showed a prevalence of LCS of 23.4% [25]. The incidence rate of LCS resulted in being different among cohorts due to study population heterogeneity, different COVID-19 severities, and the multifactorial pathogenesis of LCS [26]. The frequency of persistent symptoms after a mild COVID-19 infection was lower, ranging from 10% to 35% [27], than the aforementioned prevalence described in the studies including mostly hospitalized COVID-19 patients [23,24]. Indeed, our prevalence of LCS was similar to that observed in outpatients with mild COVID-19.

In our study, we observed that nasal congestion and diarrhea were the most frequently reported symptoms of LCS. Differently, Lopez-Leon [3] found that neuropsychiatric disorders were the most prevalent symptoms in children with LCS and mood symptoms (16.5%), fatigue (9.7%), sleep disorders (8.4%), and headache (7.8%) resulted in being the most frequent clinical conditions. Only 7.5% and 1.7% of subjects complained of nasal congestion and diarrhea. Huang et al. [24] highlighted other neuropsychiatric symptoms in their cohort study conducted among a population of 1733 patients who reported fatigue and muscle weakness (63%) as the most frequent persistent symptoms, followed by sleep alteration (26%), anxiety, and depression (23%).

Zheng et al. [25] showed that dyspnea was the most frequent symptom of LCS among 12,424 children and adolescents (22.7%). In contrast, Funk et al. [21], in their study, reported that the most common symptoms of LCS were respiratory and systemic, particularly fatigue or weakness, which were the most presented individual symptoms.

Kikkenborg Berg et al. [11], in their cross-sectional study conducted among a population of 24,315 patients, 6630 of whom had tested positive for SARS-CoV-2 in the past, identified other persistent symptoms. In fact, among the 2997 people who reported persistent symptoms, the most frequent were headaches, tiredness, loss of appetite, and breathing difficulties.

Similarly, we found similar prevalence of fatigue (21.2%), abdominal pain (10.1%), and cough (8.1%).

Different follow-up times could partly explain the heterogeneity of the prevalence of LCS symptoms in several studies [25,28]. Indeed, the prevalence of symptoms of Long COVID, such as respiratory, cardiovascular, psychiatric, and neurological symptoms, decreased with the duration of follow-up [25]. In a prospective cohort study including 286 children (56.4%) with SARS-CoV-2 infection, Buonsenso et al. [28] observed that 1/3 of the children showed persistent symptoms 1–3 months after diagnosis, while at 6–9 months follow-up, the prevalence of children with ongoing symptoms dropped to about 1/10.

Regarding risk factors for LCS, several studies have identified an older age, female sex, pre-existing conditions, and allergic diseases as potential risk factors for the persistence of symptoms after acute COVID-19 [3,16,29,30].

In our study, we observed that females, adolescents, and Caucasians had a higher risk of developing LCS than males, school-aged children, and other ethnicities, although this was not statistically significant. Female sex was identified as a risk factor for LCS in adults [16,27,31,32], but not in children [28,30].

Additionally, we found no association between older age and LCS development. This contrasting finding could be due to our small sample size.

According to several studies in the literature [3,16,25], we also observed that the concurrent presence of allergy, respiratory, gastrointestinal, and rheumatologic diseases was a risk factor for LCS.

Osmanov et al. [18] found that the most frequent pre-existing comorbidity was food allergy (13%), followed by allergic rhinitis and asthma (9.7%), gastrointestinal (9.3%) and neurological (8.8%) diseases, and eczema (8.8%).

Zheng et al. [25] observed that an older age, female sex, poor physical or mental health, or severe infection or more symptoms represented risk factors for LCS in children.

Several studies have reported different risk factors for LCS in the adult population. For example, Notarte et al. [16] showed in their study that diabetes, obesity, and respiratory disease are the most frequent conditions that can lead to LCS.

Some authors have tried to identify protective factors for LCS. For example, Ashkenazi-Hoffnung et al. [33] found that, in children, factors that may lead to a milder severity of COVID-19 and even LCS were fewer comorbidities, vigorous immune response, reduced expression of angiotensin-converting enzyme-2 receptors, and strong thymic function that may lead to more T cells in the blood system.

Regarding acute COVID-19 symptoms, we found that fatigue, muscle, and abdominal pain were associated with LCS. A recent meta-analysis of 1,680,003 COVID-19 patients showed that the presence of fatigue and muscle pain in the acute phase was a risk factor for LCS [24]. Castanares-Zapatero et al. [34] hypothesized that persistent fatigue could be related to mechanisms that determined alterations in the central nervous system, such as cerebral hypometabolism or gliolymphatic system dysfunction.

Similarly, Morello et al. [30], in their prospective cohort study, observed that the most reported persistent symptom was fatigue (13.1%), followed by dyspnea on exertion (6.2%), headache (5.6%), and gastrointestinal symptoms (4.5%).

Children with more severe or multiple symptoms of COVID-19, or with longer hospitalization, were more likely to develop LCS [25]. The severity of the acute infection and the hospitalization were not evaluated in our analysis, as the study population consisted of outpatients with mild COVID-19.

We observed that vaccine administration was not associated with LCS development. Similarly, Morello et al. [30], in their prospective study including 1243 children aged 7.5 years, did not find a strong protective effect of partial or complete vaccination on LCS. On the contrary, a recent meta-analysis involving 629,093 adults demonstrated that two-dose vaccination before the acute infection was associated with a lower risk of LCS compared with no vaccination or one-dose vaccination [35].

The impact of vaccination on the risk of developing Long COVID in children is still unclear. Since vaccination reduced COVID-19 severity in children [35,36], we expected a lower prevalence of LCS in vaccinated children than in those unvaccinated. However, the small size of our sample and greater adherence to the vaccine among older children, who are more affected by LCS, may explain this result. Furthermore, no information on vaccination timing and small sample size could explain our findings.

However, high-quality studies are needed to better investigate this association in children.

Understanding the role of vaccinations in reducing the long-term effects of the infections, like LCS, could be useful in promoting vaccinations in hesitant young people and families.

Multisystem inflammatory syndrome in children (MIS-C) and Long COVID [37,38] are both new nosological entities that represent long-term complications of COVID-19 in children, but their underlying pathophysiological mechanisms, clinical characteristics, and therapeutic management are yet to be defined.

However, higher psychological distress, dysphoria signs, and higher negative emotions about uncertainty for the future and a loss of motivation in subjects exposed to COVID-19 compared to subjects without previous COVID-19 revealed the need for urgent action health policy to strengthen the psychological, biological, and social strategic pillars for the younger generations [39]. Screening for depression during the pandemic would also have been desirable in some at-risk patient groups, such as adolescents with eating disorders [40] or specific demographic groups, such as the elderly and youth, more susceptible or resilient to the pandemic’s mental health effects [41].

The strengths of the study were the use of a platform which allowed us to obtain a large diffusion of the survey and collect data in a short time, and the presence of a control group. Additionally, to the best of our knowledge, this study is one of the few studies focused on the role of COVID-19 vaccination as a potential predictor for LCS.

However, some methodological issues need to be mentioned. First, the survey was self-administered and selection bias could have led to an overestimation of the effect sizes [42]. Second, low socioeconomic conditions or poor digital skills could have limited the responses of the self-administered survey. Third, the low overall response rate and the small sample size reduced the generalizability of our findings; among 99 children with LCS, 31.3% showed symptoms lasting more than 4 weeks from the acute infection and 68.7% presented symptoms lasting more than 12 weeks from the acute infection. Therefore, this sample characterized by children with symptoms lasting more than 4 weeks from the acute infection was less representative. However, more generally and irrespective of the survey type, typical survey response rates can range from 5% to 30% (https://www.customerthermometer.com/customer-surveys/average-survey-response-rate/, accessed on 3 September 2022). Probably a potential explanation could be that online surveys are not yet an effective method of collecting data from Italian parents and their children compared with paper questionnaires for optimizing responses. Last, no clinical evaluation on Long COVID patients was performed, as the data were anonymous.

The advantages and disadvantages of online questionnaires have been analyzed in several studies: in 2009, a study compared answers obtained in person in 2003 with answers obtained via online questionnaire in 2006. The rate of parent access to the website was comparable to the response rate for face-to-face interviews, but the number of complete responses was lower for online interviews. The least complete responses were provided by non-traditional families, immigrant parents, and less educated parents. The advantages were the low cost and the speed with which it was possible to obtain answers [43].

## 5. Conclusions

In our study, we showed that the prevalence of LCS was 25% in a group of children and adolescents living in a region of the center of Italy. In addition, we found that fatigue, muscle, and abdominal pain in COVID-19 and the presence of concurrent comorbidities were risk factors for LCS in children.

Long COVID in children represents a major burden on communities and health systems, and a better awareness of the risk factors for Long COVID in children is needed for the optimal management and implementation of preventive strategies. The growing number of studies and new data collection techniques have improved the awareness of predisposing conditions for LCS. Our findings highlight important risk factors for LCS development in children, and the need to further characterize LCS in all age groups and to invest in research for the prevention and treatment of LCS in children, to reduce morbidity and improve long-term recovery rates. However, further larger studies are needed to better investigate which risk factors could be related to LCS in the pediatric population.

## Figures and Tables

**Table 1:** Associations of demographic characteristics and concurrent comorbidities with Long COVID syndrome.

	All Participants	Non LCS Participants	LCS Participants	OR (CI 95%)	*p* Value
	*n* = 396	*n* = 297	*n* = 99		
Gender (%)					0.909
Male	194 (49.0)	146 (49.2)	48 (48.5)	Ref.	
Female	202 (51.0)	151 (50.8)	51 (51.5)	1.03 (0.65, 1.62)	
Age (%)					0.294
6–12 years	348 (87.9)	264 (88.9)	84 (84.8)	Ref.	
>12 years	48 (12.1)	33 (11.1)	15 (15.2)	1.43 (0.72, 2.74)	
Ethnicity (%)					0.383
Non-Caucasian	35 (11.8)	28 (12.8)	7 (9.0)	Ref.	
Caucasian	262 (88.2)	191 (87.2)	71 (91.0)	1.46 (0.64, 3.82)	
COVID-19 vaccine					0.130
No	109 (44.1)	72 (48.0)	37 (38.1)	Ref.	
Yes	138 (55.9)	78 (52.0)	60 (61.9)	1.49 (0.89, 2.53)	
Doses of COVID-19 vaccine	1.98 (0.5)	1.97 (0.5)	1.98 (0.4)	1.04 (0.50, 2.18)	0.914
1					
2					
**Concurrent comorbidities**					
Neurological diseases					0.312
No	391 (99.0)	294 (99.3)	97 (98.0)	Ref.	
Yes	4 (1.0)	2 (0.7)	2 (2.0)	3.02 (0.31, 29.4)	
Gastrointestinal diseases					**<0.001**
No	381 (96.5)	292 (98.6)	89 (89.9)	Ref.	
Yes	14 (3.5)	4 (1.4)	10 (10.1)	7.97 (2.55, 30.6]	
Heart diseases					0.172
No	392 (99.2)	295 (99.7)	97 (98.0)	Ref.	
Yes	3 (0.8)	1 (0.3)	2 (2.0)	5.69 (0.46, 180.0)	
Respiratory diseases					**0.014**
No	388 (98.2)	294 (99.3)	94 (94.9)	Ref.	
Yes	7 (1.8)	2 (0.7)	5 (5.1)	7.45 (1.51, 58.70)	
Asthma					0.212
No	386 (97.7)	291 (98.3)	95 (96.0)	Ref.	
Yes	9 (2.3)	5 (1.7)	4 (4.0)	2.46 (0.57, 9.84)	
Allergic Rhinitis					
No	353 (89.4)	279 (94.3)	74 (74.7)	Ref.	**<0.001**
Yes	42 (10.6)	17 (5.7)	25 (25.3)	5.50 (2.83, 10.90)	
Atopic Dermatitis					**0.001**
No	372 (94.2)	286 (96.6)	86 (86.9)	Ref.	
Yes	23 (5.8)	10 (3.4)	13 (13.1)	4.29 (1.81, 10.50)	
Food Allergy					**0.008**
No	379 (95.9)	289 (97.6)	90 (90.9)	Ref.	
Yes	16 (4.05)	7 (2.4)	9 (9.1)	4.09 (1.40, 12.00)	
Rheumatologic diseases					**0.043**
No	392 (99.2)	296 (100)	96 (97.0)	Ref.	
Yes	3 (0.8)	0 (0.0)	3 (3.0)	21.51 (1.10, 420.12)	
Hematological diseases					0.995
No	394 (99.7)	295 (99.7)	99 (100)	Ref.	
Yes	1 (0.3)	1 (0.3)	0 (0.0)	0.99 (0.04, 24.49)	
Immunological diseases					0.080
No	393 (99.5)	296 (100)	97 (98.0)	Ref.	
Yes	2 (0.5)	0 (0.0)	2 (2.0)	15.21 (0.73, 319.50)	
Diabetes					0.178
No	394 (99.7)	296 (100)	98 (99.0)	Ref.	
Yes	1 (0.3)	0 (0.0)	1 (1.0)	9.03 (0.37, 223.49)	
Endocrinological diseases					0.178
No	394 (99.7)	296 (100)	98 (99.0)	Ref.	
Yes	1 (0.3)	0 (0.0)	1 (1.0)	9.03 (0.37, 223.49)	
Obesity					0.313
No	388 (98.2)	292 (98.6)	96 (97.0)	Ref.	
Yes	7 (1.8)	4 (1.4)	3 (3.0)	2.30 (0.42, 11.20)	
Anxiety					0.054
No	391 (99.0)	295 (99.7)	96 (97.0)	Ref.	
Yes	4 (1.0)	1 (0.3)	3 (3.0)	8.41 (0.96, 243.00)	

Data are expressed as percentages and absolute numbers and as odds ratio (OR) derived from logistic regression models; COVID-19: **COronaVIrus Disease** of 2019; LCS = Long COVID syndrome; Ref. = reference category. Bold *p* value < 0.05; *p* value obtained from Chi squared test.

**Table 2:** Associations of COVID-19 symptoms with Long COVID syndrome.

	All Participants	Non LCS Participants	LCS Participants	OR (CI 95%)	*p* Value
	*n* = 396	*n* = 297	*n* = 99		
Fever (%)					0.050
No	198 (50.0)	157 (52.9)	41 (41.4)	Ref.	
Yes	198 (50.0)	140 (47.1)	58 (58.6)	1.58 (1.00, 2.52)	
Headache (%)					0.223
No	221 (55.8)	171 (57.6)	50 (50.5)	Ref.	
Yes	175 (44.2)	126 (42.4)	49 (49.5)	1.33 (0.84, 2.10)	
Fatigue (%)					**<0.001**
No	328 (82.8)	258 (86.9)	70 (70.7)	Ref.	
Yes	68 (17.2)	39 (13.1)	29 (29.3)	2.73 (1.57, 4.74)	
Muscle pain (%)					**0.009**
No	307 (77.5)	240 (80.8)	67 (67.7)	Ref.	
Yes	89 (22.5)	57 (19.2)	32 (32.3)	2.01 (1.20, 3.34)	
Runny nose					
No	273 (68.9)	212 (71.4)	61 (61.6)	Ref.	0.074
Yes	123 (31.1)	85 (28.6)	38 (38.4)	1.55 (0.96, 2.50)	
Sore throat					0.110
No	270 (68.2)	209 (70.4%)	61 (61.6%)	Ref.	
Yes	126 (31.8)	88 (29.6)	38 (38.4)	1.48 (0.91, 2.38)	
Cough					0.108
No	289 (73.0)	223 (75.1)	66 (66.7)	Ref.	
Yes	107 (27.0)	74 (24.9)	33 (33.3)	1.51 (0.91, 2.46)	
Dyspnea					0.868
No	391 (98.7)	293 (98.7)	98 (99.0)	Ref.	
Yes	5 (1.3)	4 (1.4)	1 (1.0)	0.82 (0.03, 6.03)	
Pain during the respiration					0.997
No	395 (99.7)	296 (99.7)	99 (100)	Ref.	
Yes	1 (0.3)	1 (0.3)	0 (0.0)	0.99 (0.04, 25.58)	
Chest pain					0.799
No	389 (98.2)	292 (98.3)	97 (98.0)	Ref.	
Yes	7 (1.77)	5 (1.68)	2 (2.02)	1.26 (0.16, 6.21)	
Abdominal pain					**0.045**
No	382 (96.5)	290 (97.6)	92 (92.9)	Ref.	
Yes	14 (3.6)	7 (2.4)	7 (7.1)	3.14 (1.03, 9.59)	
Vomiting					0.235
No	367 (92.7)	278 (93.6)	89 (89.9)	Ref.	
Yes	29 (7.3)	19 (6.4)	10 (10.1)	1.65 (0.71, 3.64)	
Diarrhea					0.235
No	367 (92.7)	278 (93.6)	89 (89.9)	Ref.	
Yes	29 (7.3)	19 (6.4)	10 (10.1)	1.65 (0.71, 3.64)	
Loss of taste					0.818
No	378 (95.5)	283 (95.3)	95 (96.0)	Ref.	
Yes	18 (4.6)	14 (4.7)	4 (4.0)	0.87 (0.24, 2.54)	
Hearing loss					0.250
No	395 (99.7)	297 (100)	98 (99.0)	Ref.	
Yes	1 (0.3)	0 (0.0)	1 (1.0)	9.06 (0.37, 224.24)	
Confusion					0.311
No	389 (98.2)	293 (98.7)	96 (97.0)	Ref.	
Yes	7 (1.8)	4 (1.4)	3 (3.03)	2.31 (0.42, 11.2)	

Data are expressed as percentages and absolute numbers and as odds ratio (OR) derived from logistic regression models; LCS = Long COVID syndrome; Ref. = reference category. Bold *p* value < 0.05; *p* value obtained from Chi squared test.

## Data Availability

Data available on request due to restrictions. The data presented in this study are available on request from the corresponding author. The data are not publicly available due to privacy.

## Supplementary Materials

The following supporting information can be downloaded at: https://www.mdpi.com/article/10.3390/children11020221/s1, Table S1: General characteristics of the patients with or without previous COVID-19.

## References

[1] Fernández-De-Las-Peñas C. (2021). Long COVID: Current definition. Infection.

[2] Chen C., Haupert S.R., Zimmermann L., Shi X., Fritsche L.G., Mukherjee B. (2022). Global Prevalence of Post-Coronavirus Disease 2019 (COVID-19) Condition or Long COVID: A Meta-Analysis and Systematic Review. J. Infect. Dis..

[3] Lopez-Leon S., Wegman-Ostrosky T., Ayuzo Del Valle N.C., Perelman C., Sepulveda R., Rebolledo P.A., Cuapio A., Villapol S. (2022). Long-COVID in children and adolescents: A systematic review and meta-analyses. Sci. Rep..

[4] (2020). COVID-19 Rapid Guideline: Managing the Long-Term Effects of COVID-19.

[5] Stephenson T., Allin B., Nugawela M.D., Rojas N., Dalrymple E., Pinto Pereira S., Soni M., Knight M., Cheung E.Y., Heyman I. (2022). Long COVID (post-COVID-19 condition) in children: A modified Delphi process. Arch. Dis. Child..

[6] Istituto Superiore di Sanità “Long-COVID”. https://www.iss.it/long-covid-cosa-sappiamo.

[7] Sansone F., Pellegrino G.M., Caronni A., Bonazza F., Vegni E., Lué A., Bocci T., Pipolo C., Giusti G., Di Filippo P. (2023). Long COVID in Children: A Multidisciplinary Review. Diagnostics.

[8] Wang L., Foer D., MacPhaul E., Lo Y.-C., Bates D.W., Zhou L. (2021). PASCLex: A comprehensive post-acute sequelae of COVID-19 (PASC) symptom lexicon derived from electronic health record clinical notes. J. Biomed. Inform..

[9] Soprano C.M., Ngo R., Konys C.A., Bazier A., Salamon K.S. (2023). Post-Acute Sequelae of COVID-19 (PASC) in Pediatrics: Factors That Impact Symptom Severity and Referral to Treatment. Children.

[10] Buonsenso D., Munblit D., De Rose C., Sinatti D., Ricchiuto A., Carfi A., Valentini P. (2021). Preliminary evidence on long COVID in children. Acta Paediatr..

[11] Kikkenborg Berg S., Dam Nielsen S., Nygaard U., Bundgaard H., Palm P., Rotvig C., Vinggaard Christensen A. (2022). Long COVID symptoms in SARS-CoV-2-positive adolescents and matched controls (LongCOVIDKidsDK): A national, cross-sectional study. Lancet Child Adolesc. Health.

[12] Di Filippo P., Attanasi M., Dodi G., Porreca A., Raso M., Di Pillo S., Chiarelli F. (2021). Evaluation of sleep quality and anxiety in Italian pediatric healthcare workers during the first wave of COVID-19 pandemic. BMC Res. Notes.

[13] Wang C., Ramasamy A., Verduzco-Gutierrez M., Brode W.M., Melamed E. (2023). Acute and post-acute sequelae of SARS-CoV-2 infection: A review of risk factors and social determinants. Virol. J..

[14] De Lima J.B., Salazar L., Fernandes A., Teixeira C., Marques L., Afonso C. (2023). Long COVID in Children and Adolescents: A Retrospective Study in a Pediatric Cohort. Pediatr. Infect. Dis. J..

[15] Centers for Disease Control and Prevention, National Center for Health Statistics Long COVID—Household Pulse Survey. https://www.cdc.gov/nchs/covid19/pulse/long-covid.htm.

[16] Notarte K.I., Catahay J.A., Velasco J.V., Pastrana A., Ver A.T., Pangilinan F.C., Peligro P.J., Casimiro M., Guerrero J.J., Gellaco M.M.L. (2022). Impact of COVID-19 vaccination on the risk of developing long-COVID and on existing long-COVID symptoms: A systematic review. EClinicalMedicine.

[17] Antoniou K.M., Vasarmidi E., Russell A.-M., Andrejak C., Crestani B., Delcroix M., Dinh-Xuan A.T., Poletti V., Sverzellati N., Vitacca M. (2022). European Respiratory Society statement on long COVID follow-up. Eur. Respir. J..

[18] Osmanov I.M., Spiridonova E., Bobkova P., Gamirova A., Shikhaleva A., Andreeva M., Blyuss O., El-Taravi Y., DunnGalvin A., Comberiati P. (2021). Risk factors for post-COVID-19 condition in previously hospitalised children using the ISARIC Global follow-up protocol: A prospective cohort study. Eur. Respir. J..

[19] Asadi-Pooya A.A., Nemati H., Shahisavandi M., Akbari A., Emami A., Lotfi M., Rostamihosseinkhani M., Barzegar Z., Kabiri M., Zeraatpisheh Z. (2021). Long COVID in children and adolescents. World J. Pediatr..

[20] ISARIC (2021). https://isaric.org/document/covid-19-long-term-follow-up-study/.

[21] Funk A.L., Kuppermann N., Florin T.A., Tancredi D.J., Xie J., Kim K., Finkelstein Y., Neuman M.I., Salvadori M.I., Yock-Corrales A. (2022). Post–COVID-19 Conditions among Children 90 Days After SARS-CoV-2 Infection. JAMA Netw. Open.

[22] Wille N., Badia X., Bonsel G., Burström K., Cavrini G., Devlin N., Egmar A.-C., Greiner W., Gusi N., Herdman M. (2010). Development of the EQ-5D-Y: A child-friendly version of the EQ-5D. Qual. Life Res..

[23] Quaranta V.N., Portacci A., Dragonieri S., Locorotondo C., Buonamico E., Diaferia F., Iorillo I., Quaranta S., Carpagnano G.E. (2023). The Predictors of Long COVID in Southeastern Italy. J. Clin. Med..

[24] Huang C., Huang L., Wang Y., Li X., Ren L., Gu X., Kang L., Guo L., Liu M., Zhou X. (2021). RETRACTED: 6-month consequences of COVID-19 in patients discharged from hospital: A cohort study. Lancet.

[25] Zheng Y.-B., Zeng N., Yuan K., Tian S.-S., Yang Y.-B., Gao N., Chen X., Zhang A.-Y., Kondratiuk A.L., Shi P.-P. (2023). Prevalence and risk factor for long COVID in children and adolescents: A meta-analysis and systematic review. J. Infect. Public Health.

[26] Davis H.E., McCorkell L., Vogel J.M., Topol E.J. (2023). Long COVID: Major findings, mechanisms and recommendations. Nat. Rev. Microbiol..

[27] Van Kessel S.A.M., Olde Hartman T.C., Lucassen P.L.B.J., van Jaarsveld C.H.M. (2021). Post-acute and long-COVID-19 symptoms in patients with mild diseases: A systematic review. Fam. Pract..

[28] Buonsenso D., Munblit D., Pazukhina E., Ricchiuto A., Sinatti D., Zona M., De Matteis A., D’ilario F., Gentili C., Lanni R. (2022). Post-COVID Condition in Adults and Children Living in the Same Household in Italy: A Prospective Cohort Study Using the ISARIC Global Follow-Up Protocol. Front. Pediatr..

[29] Borch L., Holm M., Knudsen M., Ellermann-Eriksen S., Hagstroem S. (2022). Long COVID symptoms and duration in SARS-CoV-2 positive children—A nationwide cohort study. Eur. J. Pediatr..

[30] Morello R., Mariani F., Mastrantoni L., De Rose C., Zampino G., Munblit D., Sigfrid L., Valentini P., Buonsenso D. (2023). Risk factors for post-COVID-19 condition (Long COVID) in children: A prospective cohort study. EClinicalMedicine.

[31] Bai F., Tomasoni D., Falcinella C., Barbanotti D., Castoldi R., Mulè G., Augello M., Mondatore D., Allegrini M., Cona A. (2021). Female gender is associated with long COVID syndrome: A prospective cohort study. Clin. Microbiol. Infect..

[32] Fernández-De-Las-Peñas C., Martín-Guerrero J.D., Pellicer-Valero J., Navarro-Pardo E., Gómez-Mayordomo V., Cuadrado M.L., Arias-Navalón J.A., Cigarán-Méndez M., Hernández-Barrera V., Arendt-Nielsen L. (2022). Female Sex Is a Risk Factor Associated with Long-Term Post-COVID Related-Symptoms but Not with COVID-19 Symptoms: The LONG-COVID-EXP-CM Multicenter Study. J. Clin. Med..

[33] Ashkenazi-Hoffnung L., Shmueli E., Ehrlich S., Ziv A., Bar-On O., Birk E., Lowenthal A., Prais D. (2021). Long COVID in Children. Pediatr. Infect. Dis. J..

[34] Castanares-Zapatero D., Chalon P., Kohn L., Dauvrin M., Detollenaere J., Maertens de Noordhout C., Primus-de Jong C., Cleemput I., Van den Heede K. (2022). Pathophysiology and mechanism of long COVID: A comprehensive review. Ann. Med..

[35] Watanabe A., Iwagami M., Yasuhara J., Takagi H., Kuno T. (2023). Protective effect of COVID-19 vaccination against long COVID syndrome: A systematic review and meta-analysis. Vaccine.

[36] Klein N.P., Stockwell M.S., Demarco M., Gaglani M., Kharbanda A.B., Irving S.A., Rao S., Grannis S.J., Dascomb K., Murthy K. (2022). Effectiveness of COVID-19 Pfizer-BioNTech BNT162b2 mRNA Vaccination in Preventing COVID-19–Associated Emergency Department and Urgent Care Encounters and Hospitalizations Among Nonimmunocompromised Children and Adolescents Aged 5–17 Years—VISION Network, 10 States, April 2021–January 2022. MMWR Morb. Mortal. Wkly. Rep..

[37] Di Filippo P., David D., Attanasi M., Rossi N., Chiarelli F. (2023). Case report: Increased troponin level in 125 children during COVID-19. Front. Pediatr..

[38] Noval Rivas M., Porritt R.A., Cheng M.H., Bahar I., Arditi M. (2022). Multisystem Inflammatory Syndrome in Children and Long COVID: The SARS-CoV-2 Viral Superantigen Hypothesis. Front. Immunol..

[39] Ranieri J., Guerra F., Cilli E., Martelli A., Capuani A., Di Giacomo D. (2023). Psychological Distress and Negative Emotions in Post-COVID Infection: A Comparative Study of the COVID and NO-COVID Young Patients. Psychol. Rep..

[40] Akgül S., Akdemir D., Nalbant K., Derman O., Ersöz Alan B., Tüzün Z., Kanbur N. (2021). The effects of the COVID-19 lockdown on adolescents with an eating disorder and identifying factors predicting disordered eating behavior. Early Interv. Psychiatry.

[41] Chen P.J., Pusica Y., Sohaei D., Prassas I., Diamandis E.P. (2021). An overview of mental health during the COVID-19 pandemic. Diagnosis.

[42] DesRoches C.M., Campbell E.G., Rao S.R., Donelan K., Ferris T.G., Jha A., Kaushal R., Levy D.E., Rosenbaum S., Shields A.E. (2008). Electronic Health Records in Ambulatory Care—A National Survey of Physicians. N. Engl. J. Med..

[43] Heiervang E., Goodman R. (2009). Advantages and limitations of web-based surveys: Evidence from a child mental health survey. Chest.

