# Supplementary material for: Potential Predictors of Long COVID in Italian Children: A Cross-Sectional Survey
Source: Children (Basel). 2024 Feb 9;11(2):221. doi: 10.3390/children11020221 (PMC10887525; doi:10.3390/children11020221)
Supplement: Supplementary file 1 [file children-11-00221-s001.zip › children-2786645-supplementary.pdf]

Supplementary table S1. General characteristics of the patients with or without previous COVID-19.

	All pts	Pts with no previous COVID-19	Pts with previous COVID-19	P value
	n=569	n=111	n=458	
Gender (%)				0.406
Male	290 (51.0)	61 (55.0)	229 (50.0)	
Female	279 (49.0)	50 (45.0)	229 (50.0)	
Ethnicity (%)				0.454
Non-Caucasian	48 (11.5)	7 (8.5)	41 (12.2)	
Caucasian	369 (88.5)	75 (91.5)	294 (87.8)	
COVID-19 vaccine (%)				**<0.001**
No	150 (40.0)	28 (25.7)	122 (45.9)	
Yes	225 (60.0)	81 (74.3)	144 (54.1)	
Hospitalization, Yes (%)	0 (0.0)	0 (0.0)	0 (0.0)	.
Age (%)				0.178
6-12 years	496 (87.2)	92 (82.9)	404 (88.2)	
≥12 years	73 (12.8)	19 (17.1)	54 (11.8)	
Doses of COVID-19 vaccine administered	1.99 (0.4)	2.01 (0.3)	1.98 (0.5)	*0.505

Data are expressed as mean and standard deviation, absolute numbers and percentages; Pts: participants; COVID-19: COronaVIrus Disease of 2019.

Bold p value<0.05; p value obtained from Chi squared test; *p value obtained from Unpaired t test.
